# Comparison of topical ethyl chloride spray and subcutaneous 1% mepivacaine for local anesthesia in single-rod etonogestrel implant insertion: an observational study

**DOI:** 10.3389/fmed.2026.1729384

**Published:** 2026-06-26

**Authors:** Rosana Garrido-Santamaría, Jesús Martínez-Tofé, Noelia Navas-Echazarreta, Antonio Rodríguez-Calvo, Michał Czapla, Ignacio Larrayoz-Roldán, Raúl Juárez-Vela, Antonio Martinez-Sabater, Regina Ruiz De Viñaspre-Hernandez

**Affiliations:** 1 Centre for Sexual and Reproductive Health Care of La Rioja, Gobierno de La Rioja, Logroño, Spain; 2 Interuniversity Doctoral Program in Global Health by the University of Zaragoza, The University of La Rioja, The Public University of Navarre and The University of Lleida, Universidad de la Rioja, Logroño, Spain; 3 Grupac Research Group, Department of Nursing, University of La Rioja, Logroño, Spain; 4 Hospital Universitario de Salamanca, Salamanca, Spain; 5 Department of Emergency Medical Service, Faculty of Nursing and Midwifery, Wrocław Medical University, Wrocław, Poland; 6 Group of Research in HealthCare (GRUPAC), Faculty of Health Sciences, University of La Rioja, Logroño, Spain; 7 Faculty of Nursing, Universitat de València, Valencia, Spain

**Keywords:** contraceptive implant, ethyl chloride, local anesthesia, mepivacaine, Midwifery, pain, reproductive health

## Abstract

**Introduction:**

This observational study examined the association between two local anesthetic techniques—topical ethyl chloride spray and subcutaneous 1% mepivacaine injection—and pain perception during the insertion of the single-rod etonogestrel contraceptive implant (Implanon NXT ^®^). The study also compared the immediate procedural resources required for each technique, including application time and material use.

**Methods:**

A total of 144 women were randomly allocated to receive ethyl chloride or mepivacaine solely to minimize selection bias within an observational design. All procedures were performed by experienced midwives in a primary care reproductive health setting. Overall pain was assessed using a 10-cm Visual Analogue Scale (VAS) at the end of the procedure. Procedural cost, anesthetic application time and adverse events were recorded. Pre-procedural anxiety and nervousness were assessed using validated VAS measures.

**Results:**

No statistically significant differences in overall pain scores were observed between groups. Ethyl chloride required significantly less time to achieve adequate anesthesia (22.1 s vs. 64.6 s; *p* < 0.01) and had lower procedural cost estimates (€ 0.11 vs. € 0.21; *p* < 0.01). One needlestick injury occurred in the mepivacaine group. Pre-procedural nervousness was the only variable showing a significant, though weak, association with higher pain levels (rho = 0.23; *p* < 0.01).

**Conclusion:**

Ethyl chloride spray was associated with shorter anesthetic application time and lower procedural resource use without increasing perceived pain, suggesting that it may enhance procedural efficiency and support workflow optimization in high-demand reproductive healthcare settings.

## 1 Introduction

The single rod etonogestrel implant is a widely used long acting reversible contraceptive (LARC) method routinely provided in primary sexual and reproductive healthcare settings. Robust scientific evidence has consistently demonstrated its high contraceptive effectiveness, with failure rates below 1% in both perfect and typical use, as well as a favorable safety profile (1). In line with this evidence, major international and national organizations—including the World Health Organization (WHO), the International Federation of Gynecology and Obstetrics (FIGO), the Centers for Disease Control and Prevention (CDC), the Faculty of Sexual and Reproductive Healthcare (FSRH), the International Confederation of Midwives (ICM), and the Spanish Society of Contraception (SEC)—recommend LARC methods, including the etonogestrel implant, as first line contraceptive options (2–7).

The implant commercialized in Spain since 2011, Implanon NXT ^®^, contains a single flexible, radiopaque, soft, non-biodegradable rod of white or off-white color, measuring 4 cm in length and 2 mm in diameter; it only contains etonogestrel, which is released over 3 years. It is preloaded into a sterile, disposable applicator. The applicator is designed to be handled with one hand and facilitates the correct subcutaneous insertion of the implant just below the skin on the inner side of the upper arm (non-dominant).

Since June 2022, the health system in La Rioja has fully funded all healthcare costs associated with implant use.

In Spain, only 1.9% of women using contraception chose the implant (8). Fear of pain during implant insertion could be one of the factors explaining the low uptake of the implant as a contraceptive method (9, 10). For this reason, both the SEC and the Spanish Association of Medicines and Pharmaceutical Products (AEMPS) recommend anesthetizing the area for implant insertion but do not specify which local anesthesia technique to use (11, 12). The technical leaflet for the implant proposes two options: the use of ethyl chloride spray or the injection of 2 ml of 1% mepivacaine just below the skin along the planned insertion channel (12).

Cloretilo cheminosa is a product for topical use on the skin containing ethyl chloride. It belongs to the group of cryo-anesthetic drugs and, when applied as an aerosol from about 15 cm away, causes a strong and sudden drop in skin temperature, manifested by a whitened and hardened appearance. The cooling effect temporarily interrupts the pain sensation, possibly through desensitization of pain receptors or activation of ion channels involved in pain transmission. The duration of application is approximately 10 s. It is indicated prior to rapid invasive techniques and minor surgical interventions (13). Storage and use: keep away from heat, protect from sunlight, and do not expose to temperatures above 50 °C. The container is self-sealing, which allows its use over time until the expiry date of the product.

The injectable pharmaceutical form of mepivacaine contains 10 mg of hydrochloride mepivacaine per ml of injectable solution (100 mg per 10-ml ampoule). Mepivacaine inhibits nerve fiber function, reduces membrane permeability and the rapid entry of sodium ions, thereby preventing impulse generation and conduction (14). Mepivacaine injection has demonstrated its efficacy in reducing pain during minor short-duration procedures, such as cannulation, incisions, or drains (15). Storage and use: It does not require refrigeration or special temperature conditions and is available in 2 ml containers. Each container is single use; once opened, it must be used immediately, and any unused portion must be discarded.

Both pharmaceutical products have established analgesic effects and are widely available in reproductive health units; however, the choice between them often depends on practitioner preference, previous experience, or local logistics rather than robust empirical evidence. In addition, each option presents specific contraindications that must be considered in clinical decision-making. Ethyl chloride spray is contraindicated in patients with hypersensitivity to the product and should be used with caution on compromised skin or when excessive application may increase the risk of local tissue injury (16). In contrast, mepivacaine (10 mg/ml) is contraindicated in individuals with hypersensitivity to amide-type local anesthetics and requires caution in patients with hepatic or cardiovascular conditions due to the potential for systemic absorption, even at low doses (17). Despite these clinically relevant differences, the available literature remains limited and presents inconsistencies regarding their comparative performance, with scarce data on immediate procedural outcomes such as time, resource utilization, and occupational safety (10, 18, 19). Therefore, additional evidence from routine clinical practice may help inform decision-making, particularly in high-volume, publicly funded healthcare settings.

The aim of this study was to examine the association between the type of local anesthesia used for single-rod etonogestrel implant insertion—topical ethyl chloride spray or subcutaneous 1% mepivacaine—and patients’ pain perception, as well as to compare the immediate procedural resources involved, including time required and material costs.

## 2 Materials and methods

### 2.1 Design

This was an observational study designed to examine the association between two local anesthetic techniques and pain perception during single-rod etonogestrel implant insertion, as well as to describe the immediate procedural resources required (time and material use) and any adverse events affecting either patients or healthcare professionals. Although participants were allocated using a simple random method, this procedure was implemented exclusively to minimize selection bias and improve group comparability within an observational design, rather than to establish an experimental framework.

### 2.2 Context

The study was conducted at the Sexual and Reproductive Health Care Centre of La Rioja (Spain), a publicly funded primary care facility where midwives routinely perform contraceptive implant insertions and removals. Data collection took place between 1 February and 30 April 2025. All services related to implant provision and insertion are fully covered by the regional health system.

### 2.3 Participants

All women who visited the center for implant insertion between February and April 2025 were invited to participate in the study. Those who consented were allocated to one of two groups: local anesthesia with mepivacaine 1% (AM) or local anesthesia with ethyl chloride spray (AC), using a computer-generated random sequence. To minimize variability due to operator skill, all procedures were performed by two midwives with more than 5 years of experience in implant insertion and removal.

### 2.4 Inclusion criteria

Women were eligible to participate if they met the World Health Organization (WHO) medical eligibility criteria for contraceptive use (categories I–II) and provided written informed consent. For minors (age <16 years), consent was required from both the participant and a legal guardian.

### 2.5 Exclusion criteria

Women were excluded if they had a known hypersensitivity to any component of the anesthetic agents used, including ethyl chloride or amide-type local anesthetics such as mepivacaine, or presented conditions contraindicating their use, such as skin lesions at the application site or clinically relevant contraindications to local anesthetics. Additional exclusion criteria included expressed preference for one technique, needle phobia, or cognitive or linguistic barriers preventing adequate understanding of the study and provision of informed consent.

### 2.6 Variables

#### 2.6.1 Exposure variable

The exposure was the type of local anesthetic used:

Mepivacaine 1%: Administered subcutaneously via a needle at the planned incision site on the arm’s skin where the implant would be inserted.

Ethyl chloride spray: Applied topically by spraying over the area to be anesthetized.

The skin was routinely prepped with chlorhexidine 2%. In the ethyl chloride spray group (AC), the insertion site was continuously sprayed for 3 s from about 15 cm away. This was repeated once or twice more until the skin adopted a hard and whitened appearance. The anesthetic effect was almost immediate (0.5 s) and lasted for 30–60 s. In the mepivacaine injection group (AM), 1 ml of 1% mepivacaine was administered subcutaneously (2–3 mm deep) using a 24G needle at the planned incision site. This anesthetic effect was delayed by at least 30 s but persisted for more time. The implant was then inserted.

#### 2.6.2 Outcome variables

Overall pain: defined as pain during anesthetic administration plus implant insertion, measured immediately after the procedure using a 10-cm Visual Analogue Scale (VAS).

Procedural resource use, including: (1) Material cost, estimated per participant based on product consumption; for mepivacaine 1%, the cost of materials (needles, syringes) and the pharmacological dose per patient were estimated and for ethyl chloride spay, the number of sprays from a 100-gram container was determined beforehand, and the price of the sprays used per woman was estimated. (2) Procedure time, measured in seconds from initiation of the anesthetic technique to initiation of implant insertion.

Adverse events, recorded for both midwives and participants.

#### 2.6.3 Predictor variables

Sociodemographic and clinical variables were collected to characterize the sample and explore potential associations with pain perception.

Age (years); BMI (weight/height^2^); nationality (Spanish or migrant); country of origin for migrant women; use of analgesics 2 h before implant insertion (yes/no); prescription medications taken on the day of insertion (yes/no); use of drugs other than prescribed medications on the day of insertion (yes/no). Pre-procedural fear and nervousness were measured with two Visual Analogue Scales (VAS) of 10 cm, where 0- represented no symptoms (fear, nervousness) and 10- maximum intensity of fear and nervousness).

### 2.7 Data collection

Exposure and predictor variables were collected after informed consent and prior to anaesthesia administration. Outcome variables were recorded immediately following implant insertion. All data were collected prospectively using standardized forms.

### 2.8 Sample size

Using EPIDAT software, a sample size of 74 women was estimated to detect a difference of 0.5 cm on the VAS between groups, assuming equal variances, with 85% power and a statistical significance level of 0.05. The sample was expanded to 144 women randomly distributed between the AM and AC groups.

### 2.9 Statistical analysis

Variables are described according to their characteristics. Quantitative variables (age, BMI, level of fear, level of nervousness before implant insertion, and pain during insertion) were summarized using means and standard deviations or medians and interquartile ranges, depending on distribution. Categorical variables (type of anesthetic, reason for choosing the implant, nationality, country of origin, psychiatric condition, use of analgesics, drug consumption, medication intake) were presented as frequencies and percentages.

Differences in participants’ characteristics between groups (GM and GC) were assessed using the Student’s *t*-test or Mann-Whitney U test depending on normality criteria, with Fisher’s correction for the chi-square test if needed.

To measure differences between groups in pain perception, economic cost, and time taken to perform the anesthetic technique, independent mean comparisons were made using the Student’s *t*-test or Mann-Whitney U test according to normality criteria. Spearman’s rank correlation coefficient was used to estimate the relationship between pain and other potential predictor variables. Differences with categorical dichotomous variables were tested using the Mann-Whitney U test. The statistical significance level was set at *p* < 0.05. Statistical analyses were performed using SPSS version 28.0 (IBM Corp, NY, USA).

### 2.10 Ethical considerations

In Spain, the methodological, ethical, and legal evaluation of observational study protocols with medications falls under the jurisdiction of the Medicines for Human Use Ethics Committees (CEIm). This opinion is unique, binding, and recognized nationwide. This study was approved by the La Rioja Medicines for Human Use Ethics Committee (CEImLAR) (code: CEImLar EOm 148).

## 3 Results

### 3.1 Women’s characteristics

A total of 144 women participated in the study, with 73 allocated to the AM group and 71 to the AC group. The average age was 27.5 years (SD = 8.5; range 13–50) and the mean BMI was 24.8 (SD = 4.5). Overall, 51.7% (73/144) were migrants. Among the migrant population, 49 originated from South America (17 Colombian, 7 Ecuadorian, 6 Bolivian, 5 Venezuelan, 4 Argentine, 3 Honduran, 3 Peruvian, 2 Brazilian, 1 Nicaraguan, 1 Salvadoran), 12 from Africa (all Moroccan), 8 from other European countries (7 Romanian, 1 Polish) and 4 from Asia (all Pakistani).

Seven participants reported a diagnosed psychiatric condition (three anxiety disorders, three depressive disorders, one ADHD, and one bipolar disorder). On the day of insertion, eight women (5.6%) had taken an NSAID (one dexketoprofen, four ibuprofen, three paracetamol), and 27 (18.8%) were taking other prescribed medications, including antibiotics (amoxicillin, ciprofloxacin), corticosteroids (prednisone), antihistamines (ebastine), antihypertensives, vitamin or mineral supplements, oral antidiabetics and statins.

Most women sought the implant for contraceptive purposes, although nine reported therapeutic indications such as heavy menstrual bleeding or dysmenorrhoea. All participants identified as female, and none reported use of non-prescribed substances.

No statistically significant differences were observed between groups in baseline characteristics, except for migrant status, which was more frequent in the AM group than in the AC group (60% vs. 42.3%; *p* = 0.03) (Table 1).

**TABLE 1:** Characteristics of the women and evaluation of the differences between the groups according to local anesthetic.

Characteristics	All women (*N* = 144)	Group of mepivacaine (*N* = 73)	Group of ethyl chloride (*N* = 71)	*p*-value
	Mean ± SD	Mean ± SD	Mean ± SD	p^1^
Age	27.5 (8.7)	27.8 (8.5)	27.2 (8.9)	0.65
BMI	24.8 (4.5)	24.8 (4.3)	24.8 (4.8)	0.99
	*n* (%)	*n* (%)	*n* (%)	p^2^
Nationality				
- Spanish women	71 (48.6)	29 (39,7)	42 (59.2)	0.03
- Other countries (migrant women)	73 (51.4)	44 (60.3)	29 (40.8)	
o Central and South America	49 (34.0)	33 (45.2)	16 (22.5)	
o North Africa	12 (8.3)	5 (6.8)	7 (9.9)	
o Europe	8 (5.5)	4 (5.5)	4 (5.6)	
o Asia	4 (2.8)	2 (2.8)	2 (2.8)	
Use of medication				
- Yes	27 (18.8)	13 (17.8)	14 (19.7)	0.78
- No	117 (81.2)	60 (82.2)	57 (80.3)	
Use of analgesic (NSAIDs)				
- Yes	8 (5.6)	3 (4.1)	5 (7.0)	0.491 (F)
- No	136 (94.4)	70 (95.9)	66 (93.0)	
Mental illness				
- Yes	7 (4.9)	2 (2.7)	5 (7.0)	0.272 (F)
- No	137 (95.1)	71 (97.3)	66 (93.0	
Medical reason for implant insertion				
- Contraceptive effect	135 (93.8)	71 (97.3)	64 (90.1)	0.95 (F)
- Treatment of metrorrhagia	9 (6.3)	2 (2.7)	7 (9.9)	

NSAIDs, non-steroidal anti-inflammatory drugs; p^1^, *p*-value estimated of student *t*-test; p^2^, *p*-value estimated of chi-square test or Fisher’s exact test.

### 3.2 Procedural resource use and pain outcomes

As shown in Table 2, there were no statistically significant differences between the AC and AM groups in overall pain perception measured at the end of the entire procedure. However, the procedural cost estimates differed between groups (€0.11 for AC vs. €0.21 for AM; *p* < 0.01). The time required for anesthetic administration also differed significantly, being shorter in the AC group (22.1 s) than in the AM group (64.6 s; *p* < 0.01).

**TABLE 2:** Key outcomes (pain, cost and time) according to local anesthetic.

Outcomes	Group of mepivacaine (*N* = 73)	Group of ethyl chloride (*N* = 71)	
	Median (IQR**)	Median (IQR**)	p^#^
- Pain (0–10 cm)*	2 (1.5)	2 (2)	0.91
	**Mean ± SD**	**Mean ± SD**	**p^##^**
- Cost (euros)	0.21	0.11	<0.01
-Time (seconds)	64.60	22.11	<0.01

Pain* evaluate through the visual analog scale (VAS); IQR**, Interquartile is the difference between the third quartile (Q3) and the first quartile (Q1) of data distribution: p^#^, *p*-value of Mann-Whitney U; p^##^, *p*-value of Student’s test.

### 3.3 Adverse events

One adverse event was recorded: a needlestick injury sustained by a midwife in the AM group during anesthetic administration. No participant-related complications were reported.

### 3.4 Factors associated with pain

Pre-procedural nervousness was the only variable showing a statistically significant, although weak, positive correlation with pain (rho = 0.23; *p* < 0.01). No significant differences in nervousness levels were found between the AC and AM groups (Mann–Whitney U = 2726.5; *p* = 0.59).

Other variables—including fear of insertion, age, BMI, nationality, prior NSAID use and the presence of a psychiatric condition—were not significantly associated with pain during implant insertion (Table 3).

**TABLE 3:** Assessment of the association of pain with other factors likely to modify pain.

Variables	Pain		
	Spearman’s Rho		p^#^
Age	−0.08		0.34
BMI	−0.15		0.08
Fear	0.13		0.12
Nervousness	0.23		<0.01
**Variables**	**Pain (median; RIQ)**	**Mann-Whitney U**	**p^##^**
Nationality			
- Spanish women	3 (2.0)	0.35	0.68
- Other countries	3 (2.5)		
Use of analgesic (NSAIDs)			
- Yes	4 (2)	3.24	0.16
- No	3 (2.5)		
Mental illness			
- Yes	2 (2)	1.88	0.34
- No	3 (2)		

NSAIDs, non-steroidal anti-inflammatory drugs; p^#^, *p*-value of Spearman’s Rank Correlation; p^##^, *p*-value of Mann-Whitney U test.

## 4 Discussion

The results of this observational study indicate that the use of topical ethyl chloride spray, compared with subcutaneous 1% mepivacaine, was associated with shorter anesthesia application time, lower immediate material use, and the absence of sharps handling, without significant differences in overall pain perception during single-rod etonogestrel implant insertion. These findings reflect procedural efficiency rather than cost-effectiveness, as the economic data presented correspond to descriptive cost estimates rather than a formal economic evaluation. The only variable showing a statistically significant, albeit weak, association with pain perception was pre-procedural nervousness, highlighting the relevance of psychological factors in pain experience.

These observations align with previous studies reporting that anxiety and fear can intensify perceived pain (19, 20). Our findings are consistent with those of Techasomboon et al., who reported similar pain levels between ethyl chloride and mepivacaine but less discomfort during administration and shorter procedure duration with ethyl chloride (18). Bentsianov et al. also found that ethyl chloride was better tolerated during the anesthesia phase, with no differences in pain perception during implant insertion (21). However, Saechoen et al. reported greater total pain with ethyl chloride (10). In the context of implant removal, Mapaisankit et al. found that ethyl chloride reduced procedural pain more effectively (22). Taken together, these findings are consistent across different clinical contexts, suggesting that the comparative performance of ethyl chloride and subcutaneous local anesthesia may be relatively robust despite variations in procedural type, clinical setting, and study population (19–22).

The present study involved single-rod implants. Differences between studies may reflect varying levels of complexity: insertion and removal of dual-rod devices tend to require longer manipulation times, which may differentially interact with the duration of the anesthetic effect. Evidence from multicenter research by Meirik et al. shows that insertion times are significantly shorter for single-rod etonogestrel implants compared with dual-rod levonorgestrel systems (23), and similar patterns have been reported for removal procedures (24). Additionally, operators with less experience require longer procedure times than highly experienced staff (23). In our study, insertions were performed by midwives with extensive experience, likely contributing to reduced overall procedure time. The duration of the implantation procedure is clinically relevant when comparing local anesthetic methods: if the procedure extends beyond the cooling effect of ethyl chloride, pain may be higher with ethyl chloride than with mepivacaine; conversely, for brief procedures, ethyl chloride may provide pain relief comparable to or better than mepivacaine.

From a procedural efficiency perspective, our findings are consistent with those of Techasomboon et al., who reported substantially shorter anesthesia application times with ethyl chloride (25). This difference may be meaningful in high-volume clinical settings where incremental time savings can increase service capacity (26). Although our study reports descriptive cost data, these should not be interpreted as an economic evaluation. Rather, they contextualize the relative resource requirements of each technique, which may be relevant for publicly funded health systems operating under resource constraints. In Spain, healthcare expenditure reached €97,661 million in 2023 (27). Identifying clinical practices that help to contain healthcare expenditure is a strategic priority. Additionally, the avoidance of needles with ethyl chloride eliminates sharps-related risks, which remains an important occupational safety concern; for instance, studies such as that of Iglesias-Osores indicate that more than half of clinical staff have experienced sharps injuries (28).

## 5 Implications for clinical practice

For trained professionals, the rapid onset of ethyl chloride (10–20 s) appears sufficient to complete implant insertion effectively. For trainees or less experienced clinicians, the longer duration of action offered by subcutaneous mepivacaine may be advantageous, though at the cost of additional time and material use. Ethyl chloride’s needle-free application also reduces occupational risk. Its rapid, superficial anesthetic effect may have applications beyond implant insertion, including minor procedures such as cannulation, removal of superficial foreign bodies or painful dressing changes, and may be particularly acceptable for pediatric patients or individuals with needle phobia.

## 6 Strengths and limitations

This study exhibits several strengths. Random allocation within the observational design reduced selection bias and enhanced group comparability. All insertions were performed by two experienced midwives, thereby minimizing operator variability. Pain was measured using a validated instrument, and the inclusion of relevant psychological and biological variables allowed for a more precise assessment of the relationship between the anesthetic technique and pain perception. Moreover, the combined evaluation of pain, procedure time, resource use and adverse events provides a comprehensive overview of each technique’s performance. Finally, conducting the study in a real-world clinical setting strengthens its practical applicability.

This study also has several limitations that should be borne in mind when interpreting the results. Firstly, although a detailed estimate of the cost per procedure was included, the economic analysis does not constitute a formal cost-effectiveness assessment; therefore, the economic data should be understood as a descriptive comparison of resources rather than a comprehensive economic analysis. Furthermore, we only measured overall pain without distinguishing between anesthesia-induced pain and post-anesthesia insertion pain. We also did not measure perceived pain at the insertion site over time. The study design did not allow for blinding of the anesthetic method used; in some women, seeing the syringe could have increased their nervousness, potentially amplifying their pain perception. These results cannot be generalized to dual-rod implants or settings with less experience in implant insertion.

## 7 Conclusion

In this study, local anesthesia with ethyl chloride proved more efficient in terms of application time and immediate consumption of materials compared with subcutaneous infiltration of 1% mepivacaine, with no significant differences in the overall perception of pain during implant insertion. Furthermore, the use of ethyl chloride avoided the handling of needles, which may help to improve occupational safety in clinical settings with high patient demand. These findings, obtained in a real-world setting with experienced practitioners, provide useful information for optimizing local anesthesia procedures during the insertion of single-rod contraceptive implants.

## Data Availability

The raw data supporting the conclusions of this article will be made available by the authors, without undue reservation.
